# Label-free two-photon fluorescence lifetime imaging of FADH_2_ for rapid diagnosis and grading of glioma

**DOI:** 10.1117/1.JBO.31.9.095004

**Published:** 2026-09-28

**Authors:** Shiqi Wang, Yixuan Yang, Chaodong Yin, Jiaqing Guo, Yan Xu, Binglin Shen, Shijun Huang, Li Wang, Jie Chen, Shuai Ye, Zhang Hong, Jianqiang Li, Liwei Liu, Lingchao Chen

**Affiliations:** a Huashan Hospital Department of Neurosurgery, Fudan University, Shanghai, China; b Shenzhen University, College of Physics and Optoelectronic Engineering, State Key Laboratory of Radio Frequency Heterogeneous Integration, Shenzhen, China; c Beijing XingYun Digital Technology Co., Ltd., Beijing, China; d Shenzhen University, School of Artificial Intelligence, National Engineering Laboratory for Big Data System Computing Technology, Shenzhen, China; e Edan Instruments, Inc., Shenzhen, China

**Keywords:** glioma, oxidation-reduction reactions, fluorescence lifetime microscopy imaging, pathological diagnosis

## Abstract

**Significance:**

Glioma diagnosis currently relies on histopathology, which provides essential morphological information but fails to capture the dynamic metabolic heterogeneity underlying tumor progression.

**Aim:**

This study seeks to establish a label-free fluorescence lifetime imaging strategy to characterize the metabolic microenvironment of gliomas, with the goal of achieving accurate tumor grading and real-time malignancy assessment.

**Approach:**

We developed a phasor plot-based two-photon fluorescence lifetime imaging microscopy (TP-FLIM) system. This imaging approach was utilized to evaluate tumor tissues by mapping microenvironmental phenotypes across four distinct glioma subtypes. In addition, we performed longitudinal *in vivo* tracking within an orthotopic glioma mouse model to monitor metabolic changes over time.

**Results:**

This method successfully identified distinct tissue regions defined as reduced flavin adenine dinucleotide (FADH_2_) hotspots. These areas serve as specific metabolic niches that are strongly associated with collagen IV deposition. Furthermore, the longitudinal *in vivo* tracking validated the feasibility of utilizing these FADH_2_ signatures to accurately discriminate varying degrees of tumor malignancy.

**Conclusions:**

These findings establish FADH_2_ hotspots as a promising and reliable optical biomarker. The study highlights TP-FLIM as a rapid and label-free platform capable of providing intraoperative metabolic navigation, precise diagnosis, and molecular stratification for glioma.

## 1 Introduction

Gliomas are the most aggressive primary brain malignancies, characterized by a diffuse, infiltrative growth pattern that compromises both neural architecture and blood-brain barrier integrity.^1^^,^^2^ Although macroscopic imaging has advanced, it still fails to provide the granularity needed to identify WHO grades or molecular signatures within the living brain.^3^^–^^6^ Current prognostic limitations are tied to the inability to visualize subtle infiltrative margins without exogenous labels, leaving the tumor’s metabolic and structural landscape largely obscured.^7^^,^^8^ This complexity is further compounded by the spatial diversity of the tumor microenvironment, where localized metabolic subregions facilitate progression and shield the tumor from conventional therapies.

The aggressive nature of gliomas is fueled by a synergy between metabolic reprogramming and microenvironmental reconfiguration. Intracellularly, an oxidative-reductive shift prioritizes glycolytic flux and mitochondrial adaptation to meet the energetic costs of malignancy.^9^^,^^10^ This metabolic state is intrinsically linked to the architectural degradation of the brain parenchyma, characterized by disordered angiogenesis and the emergence of an aberrant collagen framework.^11^^,^^12^ This collagenous remodeling not only alters the mechanical landscape of the TME but also serves as a conduit for infiltrative growth. Identifying how these metabolic and structural components spatially converge remains an unresolved challenge, leaving a significant gap in our capacity for high-precision tumor classification.

Two-photon fluorescence lifetime imaging microscopy (TP-FLIM) has been established as a robust, label-free modality for real-time metabolic mapping, enabling the quantitative assessment of endogenous redox cofactors such as nicotinamide adenine dinucleotide (NAD(P)H) and flavin adenine dinucleotide (FAD).^13^^–^^15^ Although second harmonic generation (SHG) is the conventional gold standard for visualizing fibrillar collagen, its signal is strictly dependent on noncentrosymmetric molecular assembly, leaving certain collagen isoforms or less-organized structural states undetectable.^16^ Since collagen-induced microenvironmental reprogramming indirectly drives local metabolic alterations, TP-FLIM emerges as a unique modality to characterize the spatial interplay between metabolic flux and collagen architecture.

In this work, we developed a quantitative spatial metabolic imaging framework to resolve infiltrative glioma growth patterns and molecular subtypes. Unlike traditional histopathology, which relies solely on static tissue morphology, this approach captures dynamic metabolic heterogeneity in a label-free manner. A central innovation of the study is the identification of FADH_2_ hotspots as localized metabolic microregions characterized by short fluorescence lifetimes. Spatially resolved quantitative analysis of 65 human glioma samples revealed that these hotspots are tightly associated with dense collagen IV (ColIV) deposition. Distinct microenvironmental phenotypes were systematically mapped across four glioma subtypes, and the feasibility of using FADH_2_ to discriminate tumor malignancy was further validated through longitudinal *in vivo* tracking in an orthotopic glioma mouse model. Overall, this study demonstrates that utilizing TP-FLIM to characterize FADH_2_ hotspots establishes a potent optical biomarker, providing surgeons with a precise and noninvasive tool for intraoperative metabolic navigation and real-time diagnosis.

## 2 Materials and Methods

### 2.1 Experimental Sample

A total of 65 tissue sections obtained during glioma surgery were subjected to imaging analysis, including 10 normal cortex (N), 27 low-grade glioma-infiltrated cortex (LGG), and 28 high-grade glioma-infiltrated cortex (HGG) samples. These areas were identified intraoperatively using magnetic resonance imaging (MRI) for an MRI-guided neuronavigational system by a neurosurgeon and verified visually at the operative site. Table S1 in the Supplementary Material summarizes patient demographic information. Informed consent was obtained from each patient prior to surgery, and the study was approved by the institutional review board (ethical code: KY2022762).

### 2.2 Multiplex Immunofluorescence Staining

Initially, paraffin sections were treated with an environmentally friendly dewaxing solution and absolute ethanol. They were then rinsed with distilled water. Antigen retrieval was performed in a citric acid solution. The sections were then cooled and washed with phosphate buffer saline (PBS). Using an immunohistochemistry pen, a circle was drawn around the tissue. A 3% hydrogen peroxide solution was applied to block endogenous peroxidase activity; the sections were then washed again with PBS. The sections were blocked with rabbit serum or bovine albumin (BSA), then primary antibodies were added, and the sections were incubated overnight at 4°C. They were then washed with PBS and horseradish peroxidase-labeled secondary antibodies were added. The sections were incubated; this was followed by tyramide signal amplification (TSA) reagent incubation and PBS washes. Microwave treatment in a citric acid solution was used to remove bound antibodies and prevent drying. This process of blocking, adding primary antibodies, adding secondary antibodies, and TSA incubation was repeated three times with the second, third, and fourth primary antibodies. 4’,6-diamidino-2-phenylindole (DAPI) was used for nuclear counterstaining, followed by PBS washes. Finally, an autofluorescence quencher was applied. The sections were rinsed with running water and mounted with an antifade mounting medium. Fluorescence images were acquired using NIKON ECLIPSE C1, Pannoramic MIDI, and CaseViewer software. Table S2 in the Supplementary Material lists all the primary and secondary antibodies used in the staining process.

### 2.3 Imaging

Two-photon fluorescence lifetime imaging of the tissue samples was conducted using a multiphoton confocal microscope A1 MP (Nikon, Tokyo, Japan) coupled with a lifetime unit. A two-photon femtosecond laser (Chameleon Discovery, Coherent), tunable from 780 to 1300 nm, was integrated into the main optical unit and operated at 840 nm to specifically excite FAD. A Ti laser was synchronized with a Pulse Picker, and scanning was performed using a Galvo Scanner. Tissue imaging was carried out with a 20× objective lens (MRD70200, 0.75 NA, Nikon); signal detection utilized a photomultiplier detector based on the time-correlated single photon counting (TCSPC) method. TP-FLIM imaging employed a 520/40 bandpass filter for FAD. All TP-FLIM images were captured at a resolution of 512×512  pixels, ensuring that each pixel received at least 300 photons. Over 30 random regions of interest (ROIs) were identified per section.

### 2.4 Orthotopic Glioma Mouse Model

Six- to eight-week-old NOD-SCID mice were used to establish an orthotopic intracranial glioblastoma model. All procedures were performed under sterile conditions. Mice were anesthetized with compound Zoletil (50 to 80  μL per mouse) and maintained on a heating pad throughout surgery. After preparation of the surgical field, a midline scalp incision was made to expose the skull. The implantation site was determined according to cranial landmarks at 1 mm anterior to bregma and 2.5 mm right of the midline, corresponding to the caudate nucleus. A burr hole was manually created at the marked site using a 2 to 5 mL syringe needle, with care taken to keep the needle perpendicular to the skull surface. Drilling was stopped immediately upon penetration of the skull. Fluorescently labeled human glioblastoma cells (U87MG-LUC) were injected intracranially through the burr hole to establish the orthotopic tumor model. The cranial opening was then sealed and the scalp was repositioned. Mice were randomly assigned to the control or experimental group. Imaging was performed 4 weeks after implantation in the control group and 9 weeks after implantation in the experimental group (ethical code: C202110-01).

### 2.5 Quantification and Statistical Analysis

The mean ± standard deviation (s.d.) of measurement data was calculated from at least three independent experiments and analyzed using GraphPad Prism 9 (La Jolla, CA). Statistical significance was determined at P<0.001 (****). TP-FLIM images were analyzed using maximum likelihood estimation with SPCImage (Becker & Hickl GmbH, Germany).

## 3 Results

### 3.1 TP-FLIM Lifetime Metrics Stratify Glioma Pathological Grades and IDH Genotype

As illustrated in Fig. 1(a), we established a systematic experimental workflow to evaluate the metabolic signatures of glioma. Intraoperative glioma samples were collected and processed into frozen sections, followed by the preparation of adjacent unstained sections. These serial sections were subjected to hematoxylin and eosin (H&E) staining and TP-FLIM imaging. Subsequently, TP-FLIM analysis utilized phasor plots to extract fluorescence lifetime values and separate FADH_2_ pools, which were then cross-referenced with pathological grades.

**Fig. 1:**
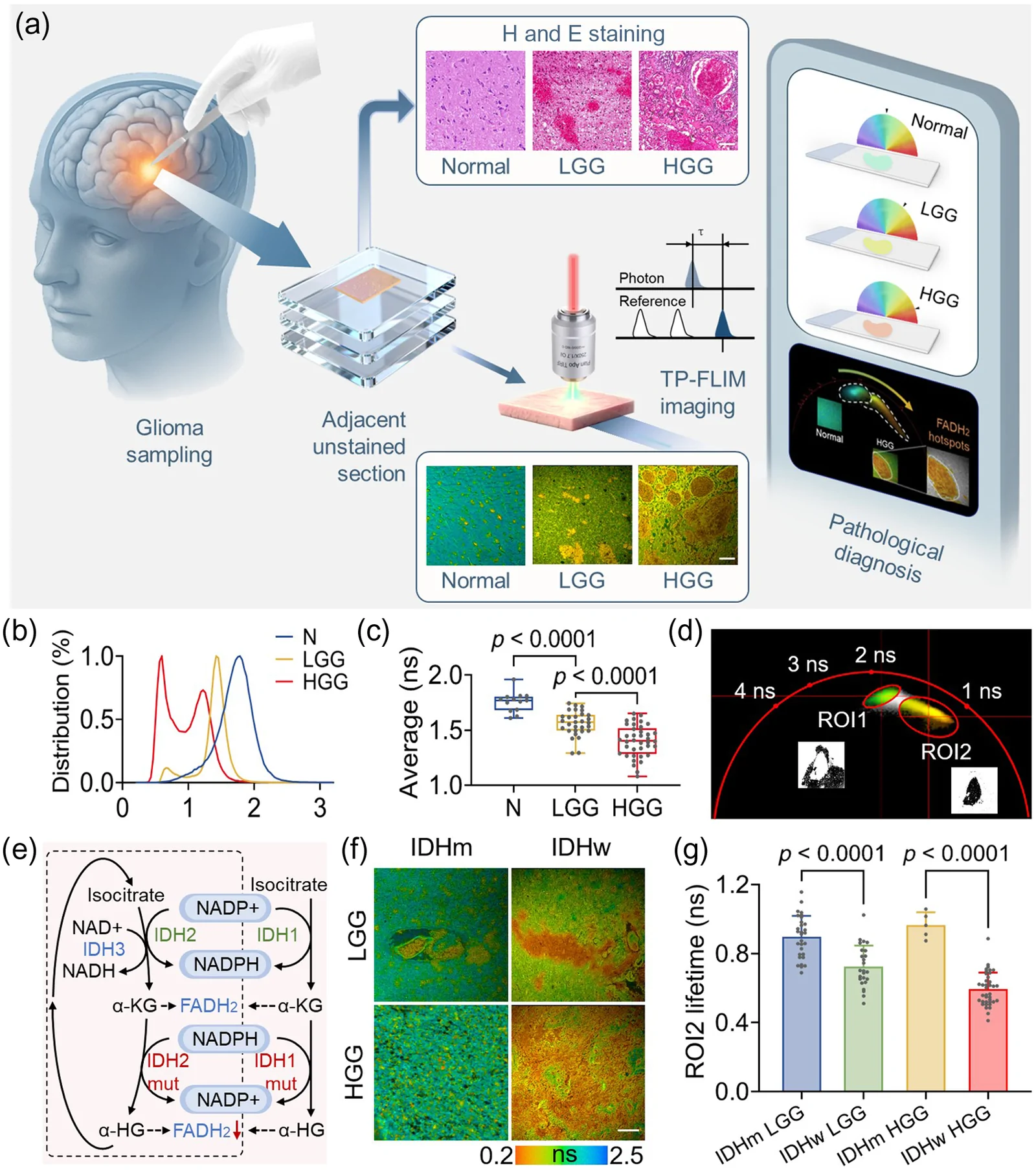
TP-FLIM lifetime analysis stratifies glioma grade and resolves IDH-associated metabolic heterogeneity through ${\mathrm{FADH}}_{2}$ hotspots. (a) Experimental workflow for bimodal analysis of intraoperative glioma samples. Scale bars, 100  μm. (b) Fluorescence lifetime distribution curves across pathological grades, showing a shift toward shorter lifetimes with increasing malignancy. (c) Quantification of mean fluorescence lifetime in N, LGG, and HGG. (d) Representative phasor plot-based segmentation defining the surrounding tissue matrix (ROI1) and short-lifetime ${\mathrm{FADH}}_{2}$ hotspots (ROI2). (e) Differential FAD expression between IDH-wildtype (IDHw) and IDH-mutant (IDHm). (f) TP-FLIM images of IDH wildtype and IDH mutant. Scale bars, 100  μm. (g) Lifetime of ROI2 in IDH wildtype and IDH mutant.

To characterize the metabolic landscape across different pathological stages, we performed TP-FLIM imaging on 65 unstained human glioma slices. The initial cohort included normal cortex (N, n=10), (LGG, n=27), and (HGG, n=28). Qualitative assessment of the TP-FLIM images revealed distinct variations in the fluorescence lifetime patterns among these groups. Quantitative analysis demonstrated a systematic shift of the fluorescence lifetime distribution curve toward shorter lifetimes with increasing malignancy [Fig. 1(b)]. As quantified in Fig. 1(c), both LGG (1.01 to 2.00 ns) and HGG (0.52 to 1.93 ns) exhibited significantly reduced average fluorescence lifetime values compared with normal cortical tissue (1.53 to 2.00 ns) (p<0.0001). To capture intratumoral heterogeneity beyond global lifetime averaging, we employed phasor-based segmentation on the TP-FLIM datasets, which allows flexible ROI assignment for selective extraction of specific lifetime components. The tissue matrix was classified as ROI1, and the short-lifetime FADH_2_ hotspots as ROI2 [Fig. 1(d)]. By distinctly visualizing the expansion of ROI2 hotspots in HGG and LGG, phasor plots establish the spatial distribution of specific fluorescence lifetime signatures as a reliable indicator for the precise grading of glioma tissues. Statistical analysis of fluorescence lifetimes demonstrated grade-dependent differences in lifetime distribution. In LGG, ROI1 and ROI2 showed overall ranges of 0.31 to 0.85 ns and 0.52 to 1.13 ns, respectively, with the densest regions confined to 0.45 to 0.58 ns and 0.70 to 0.85 ns. In HGG, the overall ranges expanded to 0.14 to 0.93 ns for ROI1 and 0.46 to 1.34 ns for ROI2, whereas the core intervals shifted to 0.45 to 0.60 ns and 0.60 to 0.80 ns, respectively.

Beyond histological grading, we investigated whether the metabolic signatures within these hotspots could provide an independent dimension for identifying IDH genotypes. IDH-mutant and IDH-wildtype tissues exhibit divergent morphological traits including cystic changes in low-grade mutant cases and prominent vascular endothelial proliferation in high-grade wildtype variants (Fig. S1 in the Supplementary Material). Figure 1(e) demonstrates distinct FADH_2_ signals between IDH-wildtype and IDH-mutant gliomas. TP-FLIM analysis revealed that IDH-wildtype gliomas consistently exhibited lower fluorescence lifetime distributions within ROI2 than IDH-mutant gliomas [Figs. 1(f) and 1(g)]. These findings demonstrate that the intrinsic lifetime signature within FADH_2_ hotspots serves as a unique metabolic indicator for classifying IDH phenotypes. This distinction establishes a robust framework for molecular subtyping based primarily on fluorescence lifetime metrics, providing a standalone diagnostic layer before the integration of additional molecular markers.

### 3.2 Collagen Deposition Spatially Defines the Structural Niche of FADH_2_ Hotspots

After characterizing the intrinsic metabolic expansion of FADH_2_ hotspots in glioma, we explored the potential role of the extrinsic tumor microenvironment in driving this heterogeneity. To determine the structural basis of these hotspots, we analyzed their spatial correlation with tumor vasculature and collagen fibers. Our initial investigations revealed a distinct absence of SHG signals within these FADH_2_ hotspots, suggesting that these specific metabolic domains are devoid of fibrillar collagen subtypes. To identify the specific collagen subtype involved, we performed immunofluorescence staining for Collagen IV (ColIV) on the identical glioma sections used for TP-FLIM, as this protein is a primary constituent of the vascular basement membrane. Figure 2(a) presents high-resolution wide-field IF images of glioma tissue alongside the corresponding TP-FLIM images. Visual inspection revealed substantial spatial colocalization between ColIV-positive regions and FADH_2_ hotspots, suggesting a potential link between FADH_2_-related metabolic features and ColIV-positive structures [Fig. 2(b)].

**Fig. 2:**
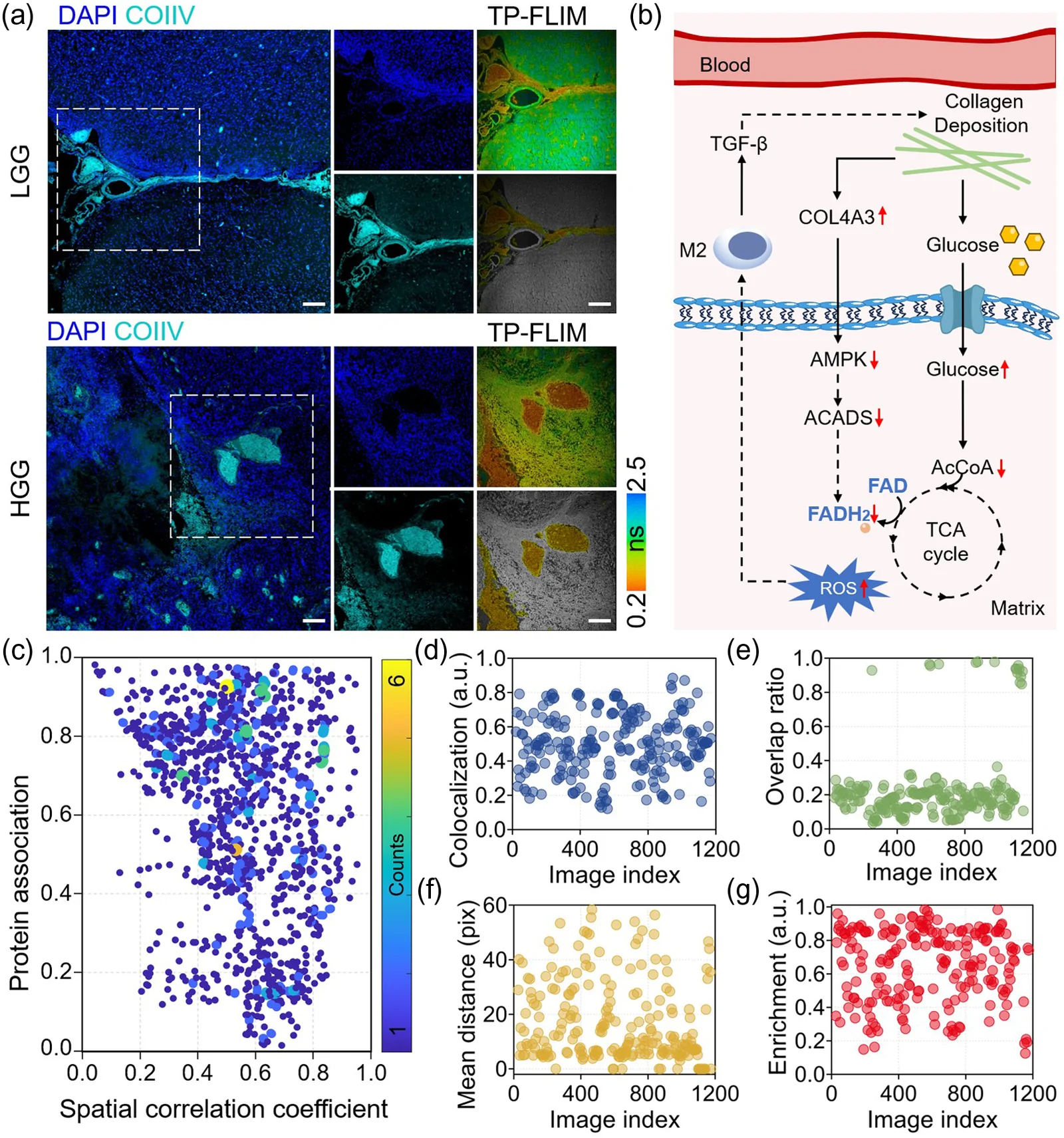
Spatial distribution of ColIV in ${\mathrm{FADH}}_{2}$ metabolic hotspots. (a) ColIV immunofluorescence staining and corresponding lifetime images in LGG and HGG. Scale bars: 100  μm. (b) Schematic diagram illustrating the proposed mechanistic link between the ColIV-rich extracellular matrix and altered intracellular redox states (${\mathrm{FADH}}_{2}$ accumulation) in glioma. (c) Quantitative colocalization analysis of ColIV and ${\mathrm{FADH}}_{2}$ with white matter integrity (WMI) in paired IF-FLIM imaging. (d)–(g) Colocalization efficiency, spatial overlap, mean intermolecular distance, and metabolic enrichment intensity analysis between COlIV and ${\mathrm{FADH}}_{2}$ paired images.

We further quantified this spatial relationship by calculating the spatial correlation coefficient between Collagen IV intensity and FADH_2_ lifetime signatures across 1200 paired imaging datasets. As quantified in Fig. 2(c), a significant correlation was observed in 70% of the sample pairs. To further characterize this association, we evaluated four key parameters: colocalization efficiency (Pearson’s r), spatial overlap (Manders’ coefficients), mean intermolecular distance, and metabolic enrichment intensity [Figs. 2(d)–2(g)]. The quantitative data demonstrated that regions with high ColIV deposition consistently corresponded to areas of shortened FADH_2_ lifetimes, confirming that Collagen IV spatially delineates the specific structural distribution of these FADH_2_ hotspots.

### 3.3 Integrated Metabolic and Microenvironmental Profiling Identifies Distinct Glioma Subtypes

To characterize the microenvironmental landscape associated with FADH_2_ hotspots, we quantified the expression of vascular and immune markers, including CD31+ for endothelial cells, IBA-1 for microglia/macrophages, and CD3+ for T lymphocytes, across different glioma grades [Fig. 3(a)]. We next assessed the spatial relationship between these markers and the collagen-defined niches. Colocalization analysis revealed distinct patterns of immune cell distribution relative to ColIV deposition [Fig. 3(b)]. Specifically, HGG tissues exhibited a markedly higher density of CD31+ vessels and IBA-1+ cells compared with both normal cortex and LGG. Quantitative correlation analysis demonstrated that the spatial overlap between IBA-1 and CD31+ was strongest in high-grade samples. By contrast, CD3+ T-cell infiltration showed no significant correlation with ColIV levels across the three groups, although a higher baseline presence of CD3+ cells was noted in normal cortical tissue.

**Fig. 3:**
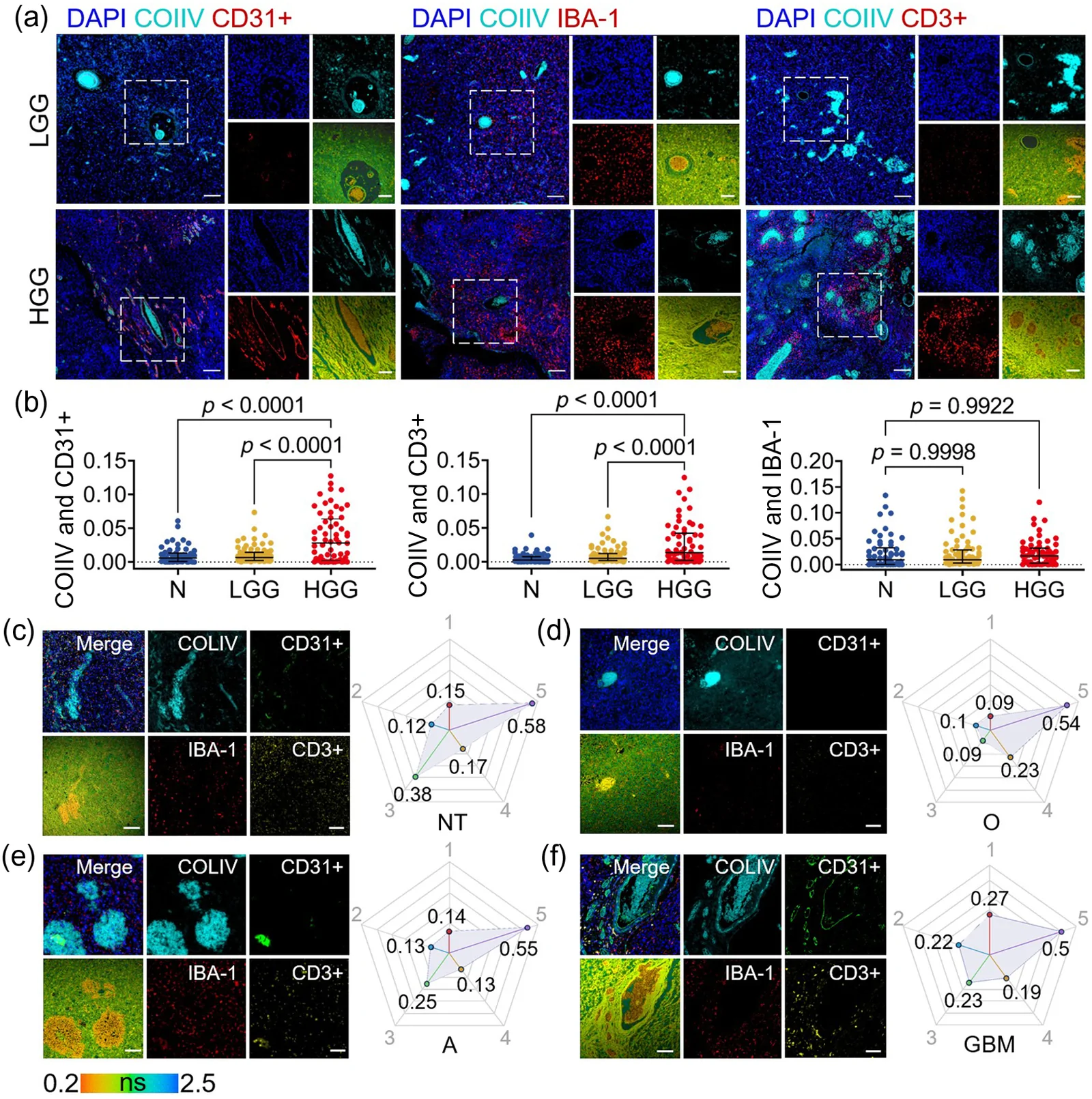
Multiparametric subtyping of glioma via integrated vascular, immune, and metabolic profiling using IF and TP-FLIM imaging. (a) Immunofluorescence staining of CD31+, IBA-1, and CD3+ with corresponding TP-FLIM images in LGG and HGG. Scale bars: 100  μm. (b) Correlation analysis among different proteins. (c)–(f) Multiparametric quantitative analysis of four distinct glioma phenotypes. Five-dimensional radar charts illustrating the normalized expression profiles of five evaluation dimensions across the four glioma subtypes, arranged in counterclockwise order from the top: CD31+, IBA-1, CD3+, ${\mathrm{FADH}}_{2}$ hotspot percentage, and ${\mathrm{FADH}}_{2}$ lifetime. Outer vertices indicate higher expression level or fluorescence lifetime. The charts integrate immune, angiogenic, and metabolic features for comparative visualization of subtype-specific phenotypes.

To systematically assess the consistency of FADH_2_ hotspot distribution across the pathological spectrum of gliomas, we extended our analysis to four representative subtypes spanning a continuous grade from low to high: low-grade neuronal tumors (WHO I-II); oligodendrogliomas (WHO II-III), typically associated with IDH1/2 mutations and 1p/19q codeletion; grade III astrocytomas, a high-grade entity with prominent cytological atypia and hypercellularity; and glioblastomas (WHO IV), the most prevalent and lethal primary brain malignancy, histologically defined by microvascular proliferation and pseudopalisading necrosis [Figs. 3(c)–3(f)]. By constructing a uniformly normalized five-dimensional radar chart, we integrated multidimensional expression information of immune infiltration, angiogenesis, and metabolic status within the tumor microenvironment, thereby achieving effective discrimination among the four molecular subtypes of glioma. The five-dimensional radar chart presents the normalized values of five evaluation dimensions in a counterclockwise order: CD31+, IBA-1, CD3+, the percentage of FADH_2_ hotspot area, and the FADH_2_ fluorescence lifetime. Each vertex of the radar chart corresponds to one evaluation dimension, and a vertex positioned closer to the outer ring indicates a higher expression level or fluorescence lifetime. By integrating multidimensional information regarding the immune microenvironment, angiogenesis, and metabolic status into a unified visualization framework, this radar chart enables an intuitive representation of the comprehensive feature profile of each sample across the five-dimensional space, thereby providing a visual basis for phenotypic comparison and classification among different glioma subtypes. Analysis of inter-subtype differences across the four glioma subtypes demonstrated that GBM harbored the highest CD31+ and IBA-1 expression levels and the shortest FADH_2_ fluorescence lifetime, all of which are in accordance with the pathological hallmarks of high-grade gliomas. The FADH_2_ hotspot proportion in GBM exceeded that of grade III astrocytomas and neuronal tumors, consistent with our earlier observations. Interestingly, oligodendrogliomas exhibited a higher hotspot percentage than GBM, a phenomenon potentially attributable to IDH1/2 mutation-induced metabolic reprogramming and the concomitant establishment of an immunosuppressive tumor microenvironment. The reduced CD3+ expression in GBM relative to low-grade tumors further corroborates its immune-evasive characteristics. Taken together, these multidimensional analyses underscore substantial microenvironmental heterogeneity across glioma subtypes, and the observed deviations from the grade-dependent trend may suggest metabolic-immune interplay driven by IDH mutations, meriting deeper exploration in future studies.

### 3.4 Longitudinal *In Vivo* Imaging Defines the Dynamic Evolution of FADH_2_ Hotspots during Glioma Progression

Building on the finding that FADH_2_ hotspots define spatially restricted malignant niches in human glioma tissues, we next investigated whether these metabolic signatures could be monitored dynamically *in vivo* throughout the course of tumor progression. To address this, we established orthotopic intracranial glioma xenograft models in immunodeficient mice and performed longitudinal TP-FLIM imaging through chronic cranial windows [Fig. 4(a)].

**Fig. 4:**
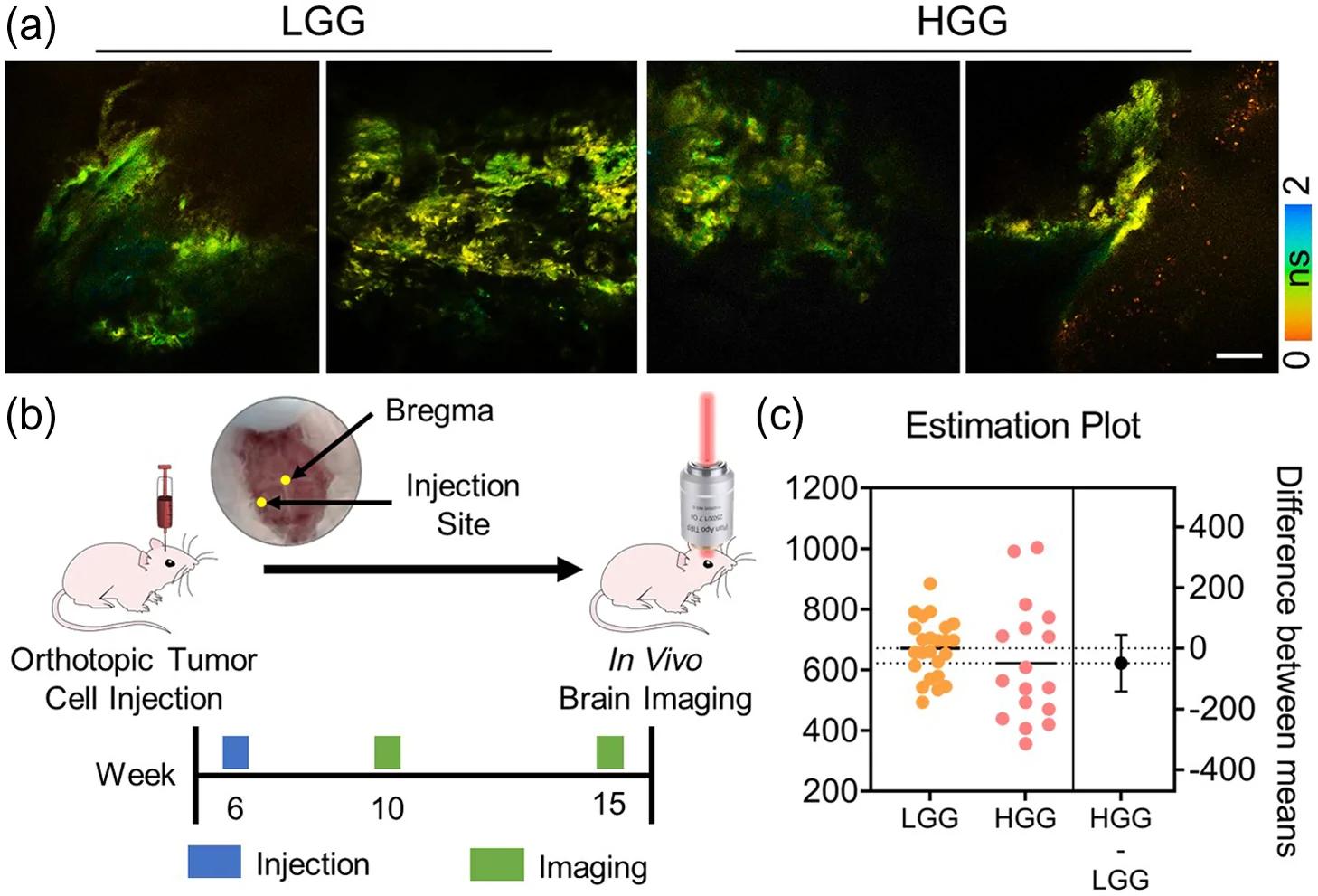
Longitudinal *in vivo* imaging validates ${\mathrm{FADH}}_{2}$ hotspots as dynamic indicators of glioma progression. (a) Representative sequential TP-FLIM lifetime maps showing the emergence and expansion of ${\mathrm{FADH}}_{2}$ hotspots during glioma progression in the live mouse brain. Scale bars, 100  μm. (b) Schematic illustration of the *in vivo* experimental workflow, including orthotopic intracranial xenograft establishment and longitudinal TP-FLIM imaging through chronic cranial windows. (c) Comparison of fluorescence lifetime distributions between early- and late-stage tumors, showing a shift toward shorter lifetimes with progression.

We subsequently tracked the temporal evolution of these metabolic signatures throughout glioma progression, where sequential *in vivo* TP-FLIM imaging revealed the spontaneous formation and progressive expansion of FADH_2_ hotspots within the murine brain [Fig. 4(b)]. These hotspot regions were characterized by markedly shortened fluorescence lifetimes and became increasingly abundant as tumors progressed. Quantitative analysis further demonstrated an increase in hotspot prevalence over time, accompanied by a progressive shift of the lifetime distribution toward shorter values [Fig. 4(c)]. Together, these findings indicate that FADH_2_ hotspots can be dynamically visualized *in vivo* and serve as a label-free metabolic biomarker of glioma progression.

## 4 Discussion

Our study identifies FADH_2_ hotspots as a label-free optical biomarker that links glioma metabolism, microenvironmental architecture, and disease progression. Using TP-FLIM, we show that spatially confined regions with short fluorescence lifetimes are not only enriched in malignant glioma tissues but also increase with pathological grade and differ according to IDH mutation status. These findings extend conventional fluorescence lifetime analysis beyond global averaging and highlight the importance of resolving intratumoral metabolic heterogeneity at the subregional level.

A major finding of this work is that the abundance of FADH_2_ hotspots correlates with glioma malignancy. Compared with normal cortex and low-grade gliomas, high-grade tumors exhibited a marked increase in hotspot prevalence together with an overall shift toward shorter fluorescence lifetimes. This progressive shortening is consistent with the metabolic reprogramming that accompanies malignant progression, in which altered redox balance and mitochondrial dysfunction reshape endogenous fluorophore behavior. Importantly, hotspot-resolved analysis further revealed distinct lifetime differences between IDH-wildtype and IDH-mutant gliomas, indicating that TP-FLIM can capture genotype-associated metabolic heterogeneity that may not be fully resolved by bulk tissue measurements alone.

Beyond their diagnostic value, our data suggest that FADH_2_ hotspots reflect a spatially organized microenvironmental state rather than a purely tumor cell-intrinsic phenomenon (Fig. S2 in the Supplementary Material). Through matched histological and immunofluorescence analyses, we found that these short-lifetime regions were preferentially associated with dense ColIV-rich matrix and CD31-positive vascular structures. This observation indicates that FADH_2_ hotspots arise within physically remodeled stromal niches, where extracellular matrix deposition and vascular abnormalities may impose metabolic stress and constrain nutrient and oxygen dynamics. Thus, the optical lifetime signature likely integrates both intrinsic metabolic rewiring and extrinsic microenvironmental pressure.

By combining TP-FLIM with multiplexed microenvironmental profiling, we further showed that metabolic heterogeneity can be interpreted within a broader ecological context. The integration of vascular, immune, and stromal features enabled the identification of distinct glioma phenotypes, suggesting that FADH_2_ hotspot patterns are embedded within subtype-specific tissue architectures. This result supports the concept that glioma metabolism should not be viewed in isolation, but rather as a dynamic property shaped by reciprocal interactions between tumor cells and their surrounding niche. From this perspective, label-free metabolic imaging provides a functional complement to histopathology and molecular classification by revealing how tumor behavior is spatially organized *in situ*.

An additional strength of our study is the *in vivo* validation of these findings in orthotopic glioma mouse models. Longitudinal TP-FLIM imaging through cranial windows demonstrated the spontaneous formation and progressive expansion of FADH_2_ hotspots during tumor growth, accompanied by a shift of lifetime distributions toward shorter values over time. Baseline histological validation further confirmed that the hotspot regions observed *in vivo* corresponded to ColIV-enriched and vascularized stromal niches identified *ex vivo*. These results establish that FADH_2_ hotspots are not static *ex vivo* artifacts, but dynamic metabolic features that can be monitored noninvasively during glioma progression.

Clinically, the identification of FADH_2_ hotspots via TP-FLIM represents a promising strategy for achieving real-time assessment of glioma tissue during clinical interventions. Unlike conventional histopathology, which depends on processed tissue and static morphological criteria, this approach provides direct optical access to metabolic states without exogenous labeling. With further technical and intraoperative validation, FADH_2_ hotspot imaging may assist in rapid glioma grading, IDH-related metabolic stratification, and the delineation of biologically aggressive regions within heterogeneous tumors.

Although our findings highlight the diagnostic utility of FADH_2_ hotspots, these results should be interpreted in light of certain limitations. Regarding the molecular origin of the signal, we have established a short-lived component as a reliable marker of malignancy, yet the specific molecular identities and conformational states responsible for these ultrashort lifetimes remain elusive. Further investigation through proteomic profiling is required to accurately correlate these lifetimes with specific enzymatic state. Nevertheless, several technical challenges remain to be addressed prior to clinical translation, including the development of a miniaturized endoscopic system to enable flexible access to surgical sites and the substantial acceleration of optical-to-pathological image conversion for real-time interpretability. Addressing these limitations will be a key focus of our future work to bridge the gap between preclinical validation and intraoperative application.

## 5 Conclusion

This study established a label-free TP-FLIM metabolic profiling framework based on quantitative vector analysis for the rapid identification and stratified evaluation of brain gliomas. This approach not only distinguished glioma tissues of different pathological grades and subtypes but also identified a spatially specific metabolic microenvironmental structure termed FADH_2_ hotspots. We found that these hotspot regions were characterized by enrichment of short-lifetime components, and that both their frequency and spatial extent increased with tumor malignant progression, thereby significantly influencing the average FAD fluorescence lifetime of the tissue. Further analyses combining TP-FLIM imaging and multiplex immunofluorescence demonstrated that FADH_2_ hotspots were closely associated with ColIV deposition and vascular-related structures, while also accompanying enhanced inflammatory responses, suggesting that they may represent an important metabolic niche through which gliomas infiltrate and expand into surrounding tissues along the perivascular microenvironment. Supported by dynamic validation in an orthotopic mouse model, these findings indicate that FADH_2_ hotspots may serve as a potential optical biomarker reflecting glioma malignancy, while TP-FLIM provides a rapid, label-free, and translationally promising strategy for intraoperative metabolic navigation and precise pathological diagnosis in glioma.

## Supplementary Material

10.1117/1.JBO.31.9.095004.s01

## Data Availability

The data that support the findings of this article are not publicly available due to ethical concerns. They can be requested from the author at wangshiqi20181@email.szu.cn.
