# Supplementary material for: Label-free two-photon fluorescence lifetime imaging of FADH2 for rapid diagnosis and grading of glioma
Source: J Biomed Opt. 2026 Sep 28;31(9):095004. doi: 10.1117/1.JBO.31.9.095004 (PMC13617214; doi:10.1117/1.JBO.31.9.095004)
Supplement: Supplementary file 1 [file JBO_031_095004_SD001.docx]

**Table S1.** Slice information of the patient demographic.

| No. | Slice number | Slice grade | Slice information |
| --- | --- | --- | --- |
| 1 | GAO-0001T | Normal | - |
| 2 | Z-00000 |  | - |
| 3 | GAO-0000-1 |  | - |
| 4 | GAO-0000N |  | - |
| 5 | LM-00001 |  | - |
| 6 | OR-00022 |  | - |
| 7 | 724N |  | - |
| 8 | 296N |  | - |
| 9 | 726N |  | - |
| 10 | 159N |  | - |
| 11 | 601N |  | - |
| 12 | 885N |  | - |
| 13 | 5312 | Low grade glioma  (LGG) | II A |
| 14 | 5422 |  | OL II |
| 15 | 5698 |  | II A |
| 16 | 5702 |  | II A |
| 17 | 6152 |  | OL II |
| 18 | 6218 |  | OL II |
| 19 | 6347 |  | A |
| 20 | 6370 |  | OL II |
| 21 | 1587 |  | A |
| 22 | 2832 |  | OL II |
| 23 | 2863 |  | Ⅰ |
| 24 | 3176 |  | OL II |
| 25 | 3458 |  | OL II |
| 26 | 4324 |  | O |
| 27 | 5090 |  | Neuroblastoma I |
| 28 | 5163 |  | I |
| 29 | 5259 |  | O |
| 30 | 27 |  | O |
| 31 | 162 |  | O |
| 32 | 262 |  | OL II |
| 33 | 313 |  | O |
| 34 | 509 |  | O |
| 35 | 668 |  | A |
| 36 | 717 |  | OL II |
| 37 | 744 |  | OL II |
| 38 | 804 |  | GG |
| 39 | 0660 |  | O |
| 40 | 5862 | High grade glioma  (HGG) | AA |
| 41 | 5930 |  | AO |
| 42 | 6143 |  | AA |
| 43 | 1616 |  | AA |
| 44 | 1649 |  | AO |
| 45 | 1861 |  | AA |
| 46 | 1883 |  | AO |
| 47 | 2798 |  | Recurrent GBM |
| 48 | 4387 |  | GBM |
| 49 | 4514 |  | Recurrent GBM |
| 50 | 4554 |  | GBM |
| 51 | 4616 |  | A Ⅲ |
| 52 | 4643 |  | GBM |
| 53 | 4644 |  | A4 |
| 54 | 4645 |  | OL Ⅲ |
| 55 | 4695 |  | GBM |
| 56 | 4725 |  | GBM |
| 57 | 5139 |  | GBM |
| 58 | 5256 |  | A4 |
| 59 | 5492 |  | AA |
| 60 | 190 |  | AA |
| 61 | 204 |  | GBM |
| 62 | 212 |  | GBN |
| 63 | 230 |  | GBM |
| 64 | 289 |  | GBM |
| 65 | 437 |  | AO |

**Table 2.** Primary and secondary antibodies used in mIF staining.

| Primary antibody | Code | Dilution ratio | Secondary antibody | Company | Corresponding TSA |
| --- | --- | --- | --- | --- | --- |
| Anti-Collagen IV Rabbit pAb | GB115688 | 1:3000 | HRP-conjugated goat anti-rabbit IgG | Servicebio,  Wuhan,  China | 440 |
| Anti-CD31 Rabbit pAb | GB11063-1-100 | 1:1000 | HRP-conjugated goat anti-rabbit IgG |  | 488 |
| Anti-Iba1 Mouse mAb | GB12105-100 | 1:3000 | HRP-conjugated goat anti-mouse IgG |  | 546 |
| Anti-CD3 Rabbit pAb | GB11014-100 | 1:1000 | HRP-conjugated goat anti-rabbit IgG |  | 647 |

Figure S1. HE-stained images of glioma sections showing microvascular pro-liferation, indicated by yellow arrows. Scale bars: 100 μm.

**Fig. S2** Glioma cells were transfected with (a) sh-NC or (b) oe-NC plasmids using Lipofectamine 2000. Transfection efficiency was assessed at different plasmid-to-reagent ratios. A ratio of 1:1 (plasmid:reagent) resulted in a transfection efficiency of approximately 1%. Similarly, a 1:2 ratio also yielded an efficiency of approximately 1%.
